# Supplementary material for: Hydrogen Induced Etching Features of Wrinkled Graphene Domains
Source: Nanomaterials (Basel). 2019 Jun 28;9(7):930. doi: 10.3390/nano9070930 (PMC6669456; doi:10.3390/nano9070930)
Supplement: Supplementary file 1 [file nanomaterials-09-00930-s001.pdf]

nanomaterials

Supporting Information

Hydrogen induced etching features of wrinkled graphene domains

Qiongyu Li [1], Fang Li [1], You Li [1], Yongping Du [1], Tien-Mo Shih [2] and Erjun Kan [1,*]

[1] Department of Applied Physics and Institution of Energy and Microstructure, Nanjing University of Science and Technology, Nanjing 210094, China; qyli@njust.edu.cn (Q.L.); lifang@njust.edu.cn (F.L.); liyou18@njust.edu.cn (Y.L.); njustdyp@njust.edu.cn (Y.D.)
[2] Department of Mechanical Engineering, University of California, Berkeley, CA 94720, USA; tshih111@gmail.com
* Correspondence: ekan@njust.edu.cn

Received: 22 May 2019; Accepted: 10 June 2019; Published: date

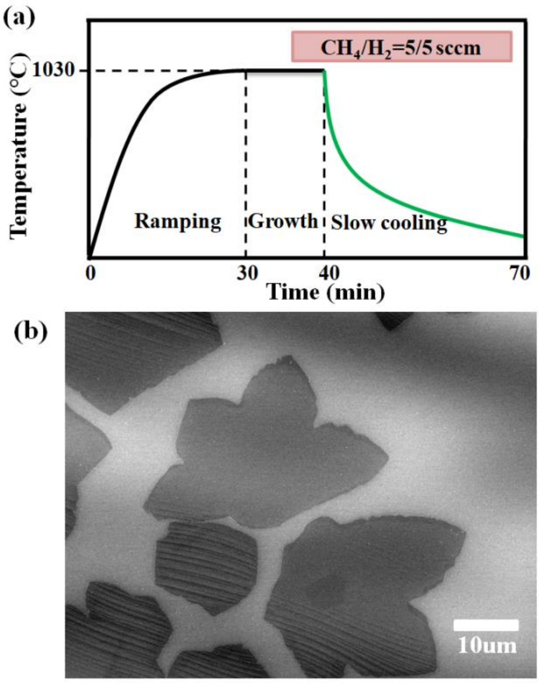

Figure S1. (a) Synthesis of graphene under slow cooling down process. (b) SEM images of as synthesized graphene domain.

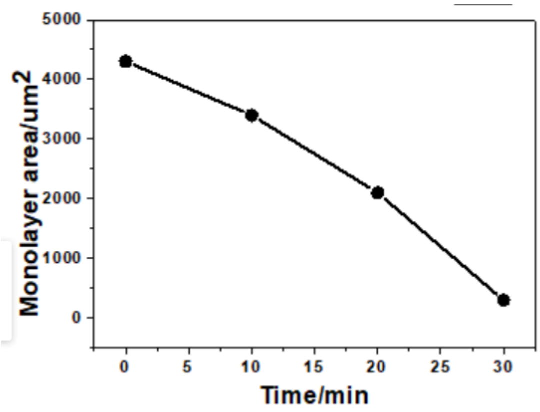

Figure S2. Statistical data of the area of monolayer graphene with etching time.
